# Pneumocephalus After Lumbar Epidural Steroid Injection: A Rare Complication With Spontaneous Resolution

**DOI:** 10.7759/cureus.73268

**Published:** 2024-11-08

**Authors:** Yasser Hegazy, Natalie N Balassiano, Ishank Gupta, Roger Stern, Muhammad Ghallab

**Affiliations:** 1 Internal Medicine, Icahn School of Medicine at Mount Sinai, Queens Hospital Center, New York, USA

**Keywords:** epidural air, epidural injections, epidural steroid injection, iatrogenic pneumocephalus, pneumocephalus

## Abstract

Pneumocephalus is a rare but potentially serious complication of spinal procedures, characterized by the presence of intracranial air. This report presents the case of a 40-year-old female who developed pneumocephalus following a lumbar epidural steroid injection. She presented to the emergency department with a persistent headache, blurred vision, and eye pain, which began shortly after the procedure. Computed tomography (CT) scans of the head and lumbar spine revealed several air pockets in the cerebellar cisterns and the left frontal horn, as well as in the epidural and paraspinal regions.

Despite the presence of intracranial air, the patient’s symptoms gradually improved with conservative management, including bed rest, caffeinated drinks, intravenous fluids, and symptomatic relief with butalbital-acetaminophen-caffeine. A repeat CT scan on day four showed a reduction in air pockets, and by day 10, all air pockets had resolved without the need for neurosurgical intervention. The patient’s headaches subsided, though she experienced mild residual vision changes.

This case emphasizes the importance of recognizing pneumocephalus as a potential complication of epidural steroid injections and highlights the efficacy of conservative treatment. While most cases of simple pneumocephalus resolve spontaneously, careful monitoring is essential to prevent progression to tension pneumocephalus, a life-threatening condition that requires urgent surgical intervention. Further studies are needed to evaluate the risks and outcomes of different techniques used during epidural procedures.

## Introduction

Pneumocephalus is a rare condition defined as the presence of gas within any intracranial compartment that can occur from numerous etiologies, e.g., barotrauma, neoplasm, spinal anesthesia, and infection. Trauma appears to be the most common documented cause of pneumocephalus, accounting for over 70% of cases [1]. Clinical presentation can include headaches, nausea, vomiting, and seizures, but it often presents as asymptomatic [1]. It is essential to distinguish between a simple pneumocephalus and a tension pneumocephalus, in which a valve mechanism allows additional air to enter the skull but prevents it from leaving, increasing pressure within it [1]. Treatment of pneumocephalus is mainly conservative, as the air within the intracranial cavity often self-resolves without further management [2].

Surgical interventions such as burr holes, needle aspiration, and bone or dural defect closure may be indicated in symptomatic pneumocephalus, tension pneumocephalus, tension pneumoventricle, and recurrent pneumocephalus or pneumocephalus persistent for more than one week [2]. Here, we discuss a case of pneumocephalus in a 40-year-old female status post-lumbar epidural steroid injection with pneumocephalus presenting with vision changes, resulting in spontaneous resolution without neurosurgical intervention.

## Case presentation

A 40-year-old female with a past medical history of hip fracture and chronic low back pain secondary to disk osteophyte complex at the L5/S1 level, for which she was undergoing physical therapy, presented to the emergency department with complaints of headache and eye pain. The headache was described as dull, persistent, of moderate intensity, and holo-cephalic. The symptoms began two days prior, immediately after she received a lumbar epidural steroid injection for her back pain. A few minutes after the lumbar injection, she developed blurry vision and constant eye pain associated with photophobia. Her symptoms persisted for over 48 hours. Before presenting to the emergency department, she experienced generalized malaise, drowsiness, fatigue, nausea, and one episode of non-bilious, non-bloody vomiting. Home medications included ibuprofen 800 mg every eight hours. She denied any personal or family history of tension or migraine headaches. Her surgical history was remarkable for left salpingo-oophorectomy due to ectopic pregnancy during her 30s. She did not smoke, drink alcohol, or use illicit drugs. She denied any fever, chills, or head trauma. A review of symptoms was otherwise unremarkable. She received Tylenol 975 mg in the emergency department, intravenous droperidol 1.25 mg, and metoclopramide 10 mg.

**Figure 1:**
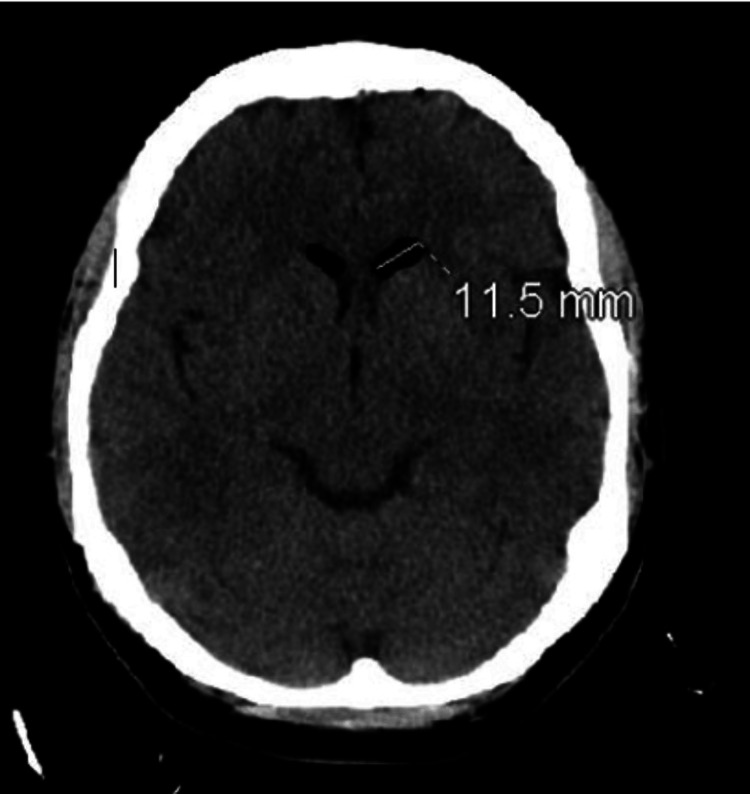
Air pocket measuring 11.5 mm in the left frontal horn.

**Figure 2:**
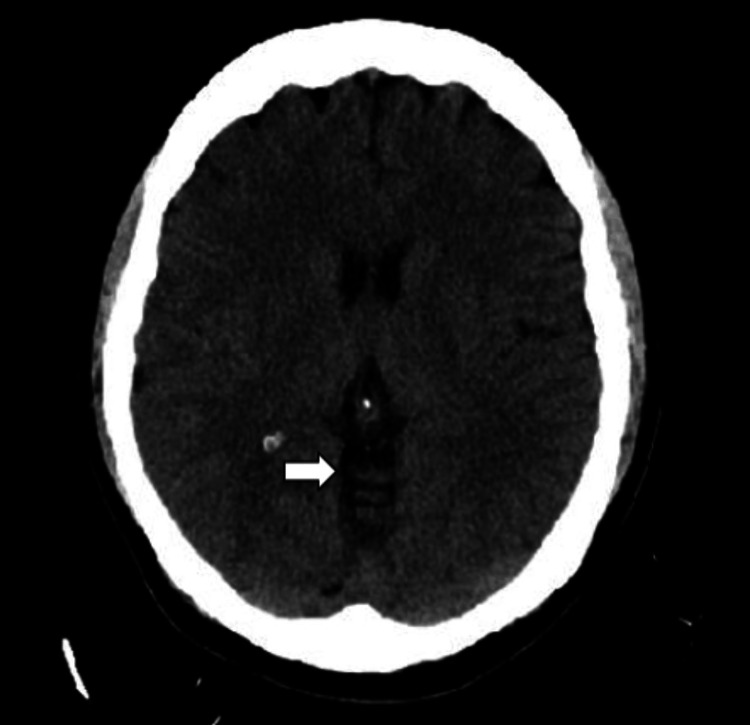
The arrow indicates several small air pockets within the cerebellar cisternal spaces.

**Figure 3:**
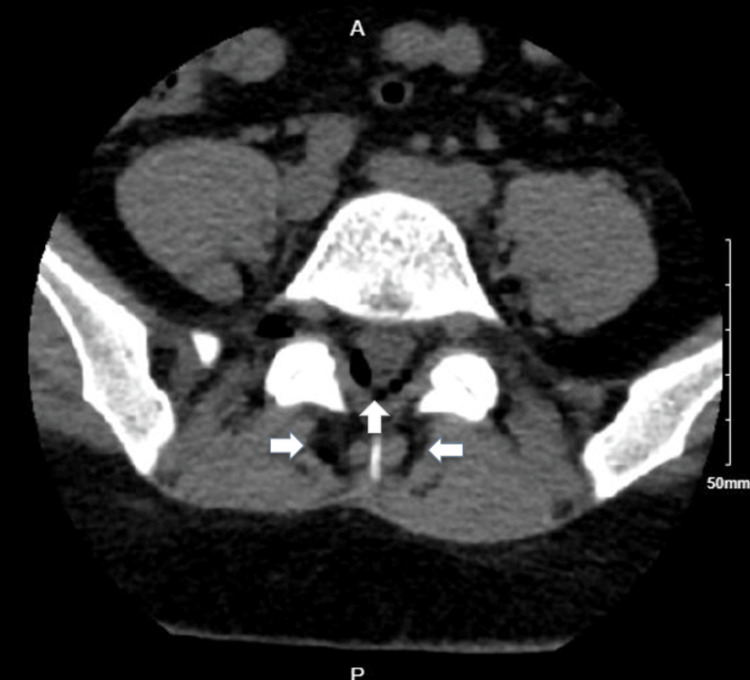
Arrows indicate several small, rounded foci of air within the epidural space and paraspinal regions.

On initial management, her vital signs were within the normal range. She was drowsy and lethargic; however, she was responsive, cooperative, and well-oriented to time, place, and person. The rest of the physical examination was unremarkable, with no focal deficits or weakness. Laboratory data was significant for a white blood cell count of 22.35 × 10^3^/µL (normal range: 4.8-10.8 × 10^3^/µL) with predominant neutrophilia, which was most likely due to the recent steroid injection. Computed tomography (CT) of the head without contrast (Figures 1, 2) demonstrated pneumocephalus with several small air pockets within the ventricles and cerebellar cisternal spaces, with the largest air pocket measuring approximately 11 mm in the left frontal horn. CT of the lumbar spine without contrast (Figure 3) demonstrated several small, rounded foci of air within the spinal canal, epidural space, and paraspinal regions.

The patient was admitted for observation, and the neurology service recommended bed rest for 24 to 48 hours, caffeinated drinks, and intravenous fluids. She was started on a combination treatment of butalbital-acetaminophen-caffeine for symptomatic relief. She had minimal symptoms over the following two days. On day four, a repeat head CT without contrast demonstrated resolution of air pockets in the cerebellar cisterns and decreased air bubbles previously seen in the frontal horns of lateral ventricles.

**Figure 4:**
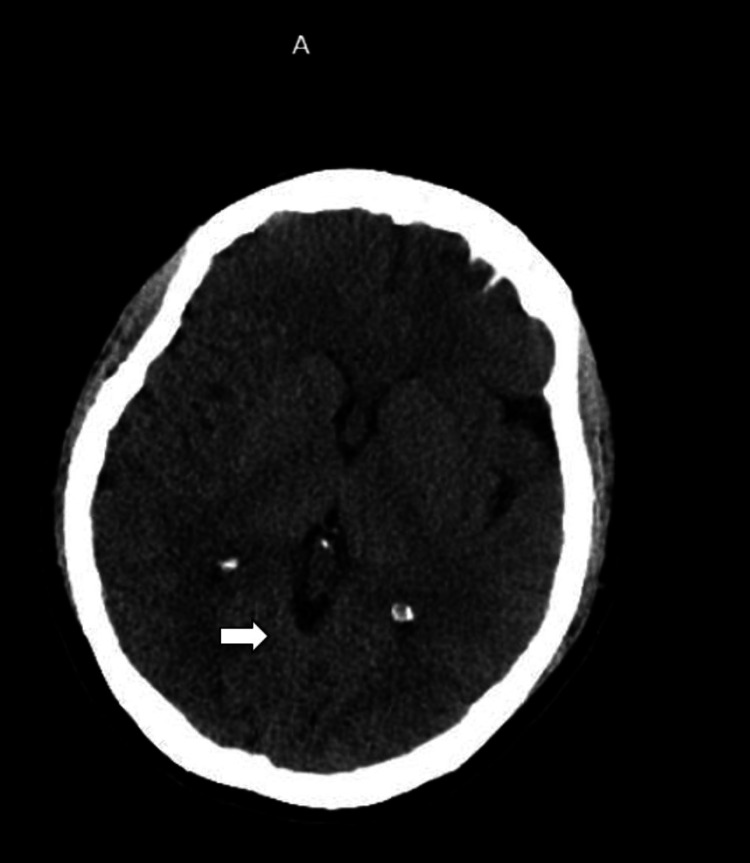
The arrow indicates the complete resolution of the air pockets within the cerebellar cisternal spaces.

**Figure 5:**
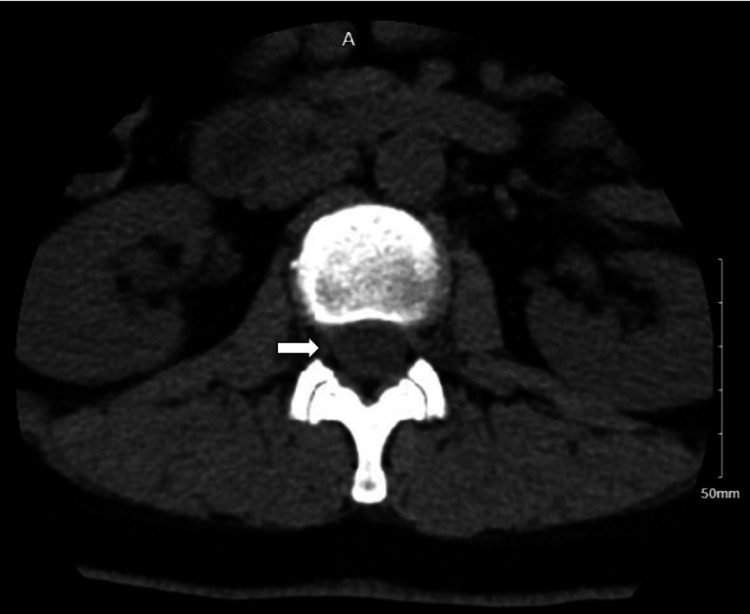
The arrow indicates the complete resolution of air within the epidural space at the level of the lumber spine.

She did not require acute neurosurgery intervention during the treatment course. She was continued on oral fluids, caffeine 300 mg/day, ibuprofen 2,400 mg/day, and as-needed butalbital-acetaminophen-caffeine combination tablets. On day 10, a non-contrast CT scan of the head (Figure 4) and lumbar spine (Figure 5) demonstrated complete resolution of pneumocephalus and the air within the spinal canal. She reported no headaches but complained of diminished vision. Her recovery was uneventful, and she was discharged after her symptoms improved.

## Discussion

The application of lumbar epidural anesthesia is associated with various complications, including, but not limited to hypotension, post-dural puncture headache, weakness, neurological defects, and, rarely, pneumocephalus [3]. Symptoms following a dural puncture typically manifest late; thus, observation of a patient’s post-dural puncture is standard to safe practice [3]. Pneumocephalus and dural puncture commonly cause headaches, although the onset of headache after spinal or epidural anesthesia is typically immediate, while dural puncture headaches usually take one to three days to erupt after the procedure. Diagnosis of simple pneumocephalus is based on clinical suspicion and can be identified by brain CT. A plain X-ray of the skull can also be used to make the diagnosis. Radiological signs used to describe findings of air within the cranium are the Mount Fuji sign, the air bubble sign, and the peaking sign [3]. Treatment of a simple pneumocephalus is conservative management with analgesics and antiemetics, supine positioning, and 100% supplemental oxygen, as the resorption of air can take around two days [1,3,4], as seen in our patient and reported by Dabdoub et al. who reported a case of a 74-year-old male status post-left frontal craniotomy for meningioma of the olfactory groove who developed pneumatocele, treated with conservative management and continuous oxygen for five days [2]. Another example supporting conservative management is the case by Gorissen et al., who discussed a case of a six-year-old male status post-deep penetrating paravertebral lumbar trauma initially treated with L4-L5 fusion with pedicle screw fixation. Subsequently, he developed postoperative pneumocephalus. He was placed in Fowler’s position. Day one postoperative imaging showed a significant reduction in pneumatocele, and day 10 postoperative imaging showed near-resolution of the pneumatocele [4]. Patients should be evaluated for signs and symptoms of elevated intracranial pressure, which may indicate the development of tension pneumocephalus. This condition requires immediate evacuation with neurosurgical decompression and repair of the initial insult, which our patient did not need. This is supported by the case reported by Sawada et al., who reported a 48-year-old male status post-right skull fracture following a fall off the stepladder, treated with cranioplasty using a titanium plate. Pneumatocele was diagnosed after the patient developed deterioration of mental status one day postoperatively, requiring treatment with emergency skin flap re-opening and insertion of epidural drain between the titanium plate and skull edge. The surgical intervention resulted in the resolution of pneumocephalus, and the patient was discharged on day 27 following cranioplasty [5]. Another case that required surgical intervention for the treatment of the pneumatocele is the case reported by Coughlan et al., who discussed a case of a 70-year-old male with minor head injury from assault who developed pneumatocele and bilateral acute on top of chronic subdural hemorrhage, treated with bilateral craniotomy and evacuation. Postoperative imaging at two and six weeks showed a significant reduction in pneumocephalus and subdural hemorrhage [6].

Epidural anesthesia is based on the successful localization of epidural space based on the loss of resistance to air or saline as the tip of the epidural needle pierces the ligamentum flavum, which can be followed by the contents of the syringe entering the epidural space. Uncommonly, penetration of the dura mater can occur when attempting to identify the epidural space, and, subsequently, if loss of resistance to air technique is being attempted, a small amount of air can be pushed into the subdural space and can be communicated into the intracranial space. Pneumocephalus, when caused by a dural puncture, is associated with the loss of resistance to air for the identification of the dural space. Some randomized clinical trials investigated the performance between loss of resistance to air vs. a loss of resistance to saline techniques, and some suggest that a loss of resistance to saline may be superior to a loss of resistance to air. However, many studies indicate no significant difference in the success of block between saline and air [7-10]. Further studies should be conducted to highlight and study the complications associated with each technique.

## Conclusions

This case emphasizes the critical need to recognize pneumocephalus as a potential complication of epidural steroid injections and highlights the effectiveness of conservative management. Although most cases of simple pneumocephalus resolve spontaneously, vigilant monitoring is essential to prevent progression to tension pneumocephalus, a potentially life-threatening condition requiring urgent surgical intervention. The case also highlights the need for further investigation into the risks associated with the loss of resistance technique during epidural procedures, particularly with air versus saline.
